# Extracellular Vesicles from Mycoplasmas Can Penetrate Eukaryotic Cells In Vitro and Modulate the Cellular Proteome

**DOI:** 10.32607/actanaturae.11506

**Published:** 2021

**Authors:** A. A. Mouzykantov, E. V. Rozhina, R. F. Fakhrullin, M. O. Gomzikova, M. A. Zolotykh, O. A. Chernova, V. M. Chernov

**Affiliations:** Kazan Institute of Biochemistry and Biophysics, FRC Kazan Scientific Center of RAS, Kazan, 420111 Russia; Kazan Federal University, Kazan, 420008 Russia

**Keywords:** mycoplasma, vesicles, internalization, human fibroblasts, proteome

## Abstract

The extracellular vesicles (EVs) produced by bacteria transport a wide range of compounds, including proteins, DNA and RNA, mediate intercellular interactions, and may be important participants in the mechanisms underlying the persistence of infectious agents. This study focuses on testing the hypothesis that the EVs of mycoplasmas, the smallest prokaryotes capable of independent reproduction, combined in the class referred to as Mollicutes, can penetrate into eukaryotic cells and modulate their immunoreactivity. To verify this hypothesis, for the first time, studies of in vitro interaction between human skin fibroblasts and vesicles isolated from Acholeplasma laidlawii (the ubiquitous mycoplasma that infects higher eukaryotes and is the main contaminant of cell cultures and vaccines) were conducted using confocal laser scanning microscopy and proteome profiling, employing a combination of 2D-DIGE and MALDI-TOF/TOF, the Mascot mass-spectrum analysis software and the DAVID functional annotation tool. These studies have revealed for the first time that the extracellular vesicles of A. laidlawii can penetrate into eukaryotic cells in vitro and modulate the expression of cellular proteins. The molecular mechanisms behind the interaction of mycoplasma vesicles with eukaryotic cells and the contribution of the respective nanostructures to the molecular machinery of cellular permissiveness still remain to be elucidated. The study of these aspects is relevant both for fundamental research into the "logic of life" of the simplest prokaryotes, and the practical development of efficient control over hypermutable bacteria infecting humans, animals and plants, as well as contaminating cell cultures and vaccines.

## INTRODUCTION

It is unclear what are the molecular mechanisms underlying the interaction between mycoplasmas (the smallest prokaryotes capable of independent reproduction combined into the class Mollicutes) and the eukaryotic cells ensuring the persistence of infectious agents [1]. Thus far, it has been established that the interaction of micro- and macro-organism cells is mediated by EVs, which carry a broad range of compounds: among those, proteins, DNA, and RNA (including short RNAs) [2]. Bacterial EVs can penetrate into eukaryotic cells and modulate immunoreactivity; this ability has been well established in some of the pathogens of persistent infections. The EVs of mycoplasmas have been described [3, 4], but no evidence is available yet regarding their ability to penetrate into eukaryotic cells.

The present study addresses the ability of EVs from A. laidlawii, ubiquitous mycoplasma infecting higher eukaryotes and the main contaminant of cell cultures and vaccine preparations, to penetrate into eukaryotic cells cultured in vitro and modulate the cellular proteome.

## EXPERIMENTAL SECTION

**Cell cultures**

A A. laidlawii PG8B culture in the middle of its growth log phase and a primary culture of human skin fibroblasts (HSF – **H**uman **S**kin **F**ibroblast) were used. The fibroblasts were obtained from skin biopsies and cultured in an αMEM medium supplemented with 100 U/mL penicillin, 100 µg/mL streptomycin, 10% bovine serum and 2 mM L-glutamine at 37°C and 5% CO_2_. Human skin samples were collected in accordance with the protocol of the experiment approved by the Expert Commission on Biomedical Ethics of the Kazan Federal University and the Republican Clinical Hospital (No. 218, November 15, 2012). Written informed consents were obtained from donors.

**Isolation of extracellular vesicles**

EVs from A. laidlawii were isolated as described in ref. [3]. The isolated vesicles were analyzed using transmission electron microscopy and scanning electron microscopy as described in ref. [3]. Microvesicles produced by HSFs in the presence and absence of A. laidlawii vesicles were isolated according to ref. [5]. The culture medium of the control and experimental HSF cultures was collected. Cells and debris were removed by centrifugation (1,500 g, 10 min). The supernatant was centrifuged at 100,000 g for 70 min (MLA-80 rotor, Beckman Coulter). The precipitates were resuspended in PBS and centrifuged at 100,000 g for 70 min. The washed precipitates were re-suspended in PBS, layered on an Optiprep density gradient (10-20-30-40-45%), and ultracentrifuged at 100,000 g for 17 h. Fractions were selected, washed three times to remove Optiprep, suspended in PBS, and stored at 4°C before analysis. The EVs from A. laidlawii were added to HSF in an amount of 100 µg (on the basis of total protein) and incubated for 4 h. The fibroblast cultures with and without EVs corresponded to the experiment and control, respectively. Vesicular DNA of A. laidlawii was detected by PCR, as described in ref. [6].

**Confocal microscopy analysis**

The preparations of EVs from A. laidlawii were stained with DiI, acridine orange and Hoechst 33342 to visualize the membrane, RNA and DNA, respectively. Preparations of HSF microvesicles were stained with anti-p53 antibodies conjugated to Alexa Fluor 647 and DiO for membrane imaging. The unbound dye molecules were removed using a concentrator with a cut-off limit of 3 kDa. The preparations were examined using a Carl Zeiss LSM 780 confocal laser scanning microscope.

To visualize the internalization of the EVs from A. laidlawii, the fibroblasts were cultured on cover glasses. EVs from A. laidlawii were stained with fluorescent dyes as described above and added to HSF. Fibroblasts were washed with buffer and fixed with 2.5% glutaraldehyde. Cell nuclei were stained with DAPI, and F-actin was stained with antibodies conjugated to Alexa Fluor 488. Slides were examined under a microscope; the data were analyzed using the ZEN 9.0 software.

Dark-field microscopy images of the stained HSF and EVs from A. laidlawii were obtained using an Olympus BX51 microscope [7]. The data were analyzed using the Exponent 7 software.

**Enzyme immunoassay**

For quantitative determination of cytokines, HSF cells were removed by centrifugation; the concentrations of interleukins (IL-6 and IL-8) in the supernatant were determined by ELISA (Vector-Best, Russia) according to the manufacturer’s protocol.

**Proteomic analysis**

Proteomic analysis of HSF was performed according to ref. [8]. The cells were detached from the plastic using trypsin and washed three times using PBS to remove the nutrient medium. The cell precipitates were dissolved in a buffer (8 M urea, 2 M thiourea, 16.7% solution (30% CHAPS + 10% NP-40)) and treated with a mixture of nucleases (Micrococcal Nuclease Mix). The protein concentration in the samples was measured by the Bradford method. The proteins were stained with CyDye DIGE Cy3 (control) and CyDye DIGE Cy5 (experiment) dyes; the reaction was stopped with a 10 mM lysine solution. The staining effectiveness was tested using 1D electrophoresis in PAAG and gel scanning on a Typhoon Trio scanner. The samples were combined (equal amounts of each sample were collected), and dithiotreitol (DTT) up to 80 mM and ampholites 3-10 up to 0.2% were added and separated using 2D electrophoresis. The gels were scanned on a Typhoon Trio scanner. The gels were stained with silver nitrate to visualize protein spots.

The protein spots on the gels were analyzed using the PDQuest v.8.01 software (Bio-Rad). The spots in which the ratio between the protein contents in the control and experiment was higher than 1.5 were cut out. The gel pieces were washed in a 1 : 1 mixture of acetonitrile : 200 mM NH4HCO3 and then incubated with DTT and iodoacetamide. The gels were dehydrated using acetonitrile. A Trypsin working solution was added to the gel and incubated for 60 min at 4°C. Tryptic digestion was performed at 37°C overnight. Peptides were extracted using a 0.5 TFA solution. Protein identification using the Ultraflextreme MALDI-TOF/TOF mass spectrometer was carried out according to the protocol [8]. The peptide samples were mixed with a matrix solution (1% 2,5-dihydroxybenzoic acid, 20% acetonitrile, 0.5% TFA), applied to the target, and air dried. Mass spectrometry was performed in the positive ion mode in the range of 500•4000 Da. The accuracy of monoisotopic mass measurements after recalibration on the basis of the peaks of trypsin autolysis was 0.007%, with allowance for the possible oxidation of methionine residues and modification of cysteine residues with acrylamide. The proteins were identified using the Mascot software in the Peptide Mass Fingerprint mode (Matrix Science) and the UniProt database. Protein identification was considered reliable (p < 0.05) at scores ≥ 44.

The DAVID database (The Database for Annotation, Visualization, and Integrated Discovery) was used for the functional annotation of the identified proteins. The metabolic pathways and cellular processes in which these proteins participate were determined (according to KEGG); gene ontology was determined using GO (molecular function, biological process, cellular component).

**Western blotting**

Targeted quantitative determination of the proteins was carried out using western blotting. The proteins from HSF lysates were separated into PAAG and transferred to the Hybond C nitrocellulose membrane. Anti-p53, anti-HSP7C, and anti-β-actin antibodies (Sigma, USA), as well as secondary antibodies conjugated with horseradish peroxidase, were used. The membranes were incubated sequentially with primary and secondary antibodies and then stained with 3,3’-diaminobenzidine (Sigma). The gels were analyzed with the ImageJ software using β-actin as a control to normalize the signal intensity of the studied samples.

**Statistical analysis**

All the experiments were carried out in three replicas. The samples were analyzed 4 h after the incubation of HSF with mycoplasma EVs in all cases, and additionally 48 h after in the case of cytokine expression analysis. Statistical analysis was performed using the RStudio package. The values of p < 0.05 were considered statistically significant.

## RESULTS AND DISCUSSION

**Fig. 1:**
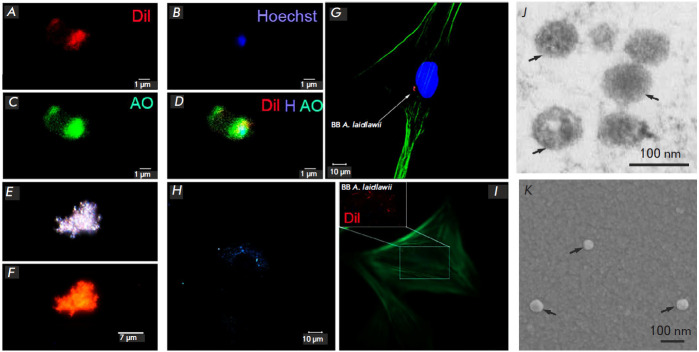
Interaction of human skin fibroblasts with EVs of *A. laidlawii*. Visualization of the purified EVs of *A. laidlawii *(*A–F, J, K*) and the ones incubated with HSF (G–I). Confocal laser microscopy (*A–D, G*): (*A*) DiI tracer (vesicular lipid staining, red); (*B*) Hoechst (vesicular DNA staining, blue); (*C*) acridine orange (AO) (vesicular RNA staining, green); (*D*) merged images (DiI, Hoechst, AO). Dark-field (*E, H*) and fluorescence (*F, I*) microscopy: (*E*) dark-field image of the aggregate of vesicles and (*F*) with fluorescence of the DiI tracer; (*G*) fluorescence EVs of *A. laidlawii *stained with DiI (red) in the HSF cavity. The cell nucleus was stained with DAPI, actin filaments, with antibodies conjugated with Alexa Fluor 488 dye (green); (*H*) dark-field image of EVs of *A. laidlawii *in the HSF cavity; and (*I*) during the fluorescence of the DiI tracer, HSF actin filaments were stained with antibodies conjugated with Alexa Fluor 488 dye (green). Transmission electron (*J*) and scanning (*K*) microscopy of EVs of *A. laidlawii *(arrows indicate individual vesicles)

**Fig. 2:**
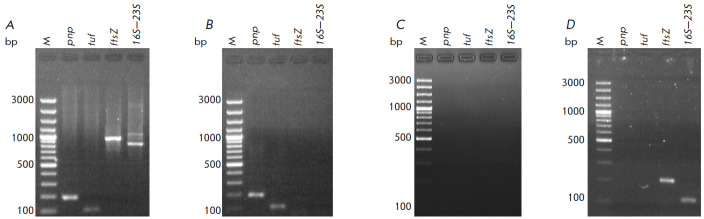
Detection of *A. laidlawii *DNA in mycoplasma EVs and HSF by PCR. DNA was extracted from *A. laidlawii *cells (*A*), EVs of *A. laidlawii *(*B*), fibroblasts (HSF) incubated without and with the EVs of *A. laidlawii *(*C*, *D*, respectively). M – DNA Ladder Marker. In PCR, the primers specific to the nucleotide sequences of the *pnp*, *tuf*, and *ftsZ *genes (encoding polyribonucleotide nucleotidyl transferase, elongation factor Tu, and cell division protein FtsZ, respectively), as well as the 16S-23S rRNA intergenic spacer region of *A. laidlawii*, were used

Previously, we showed using PCR and RNA-Seq that EVs isolated from A. laidlawii PG8B contain DNA and RNA [9]. In this work, it was found that the EVs of A. laidlawii containing RNA are able to penetrate human skin fibroblasts cultured in vitro: mycoplasma EVs are found both in the cytoplasm and in the nucleus of eukaryotic cells during their co-incubation. Figure 1 G-I shows the EVs of A. laidlawii visualized inside eukaryotic cells; Fig. 1 J, K shows photos of isolated A. laidlawii obtained by transmission electron and scanning microscopy, respectively; Fig. 2 shows the detection of DNA from A. laidlawii in fibroblasts using PCR.

Different fluorescent dyes make it possible to visualize DNA (Hoechst) and RNA (acridine orange), as well as the membrane lipids (DiI) of EVs from A. laidlawii (Fig. 1 A-C). If the object simultaneously has a lipid membrane, DNA, and RNA, then when the corresponding photos are combined, a characteristic change in the color signal is recorded due to the superimposition of fluorescence, which is observed for mycoplasma EVs (Fig. 1D).

Since the size of the vesicles in A. laidlawii (the diameter of most of them is < 120 nm) does not allow a visualization of individual vesicles using confocal fluorescence microscopy, we also used higher resolution microscopy options to analyze vesicular preparations. Thus, the images of the EVs from A. laidlawii were obtained using transmission electron (Fig. 1J) and scanning (Fig. 1K) microscopy. Individual vesicles have their characteristic morphology. They are spherical nanostructures surrounded by a membrane, their size ranging from 30 to 120 nm.

When using dark-field fluorescence microscopy, mycoplasma vesicles are recorded as aggregates (Fig. 1 E-F). Similar images were obtained for EVs from Pseudomonas aeruginosa [10]: stained with fluorescent dyes (DiO and EdU), isolated and internalized by A549 epithelial lung cells, the EVs of this bacterium were visualized in the form of clusters using confocal laser scanning microscopy. This may be due to the superimposition of the emissions of the dyes used from the adjacent individual objects. It still remains to be investigated whether this is also related to the peculiarities of the pathways for the internalization of bacterial EVs.

Short bacterial RNAs in vesicles might function as eukaryotic microRNAs and suppress translation by binding to mRNA targets [11]. This assumption was verified in model experiments with the short RNAs homologous to tRNAMet contained in P. aeruginosa vesicles. The interaction of the corresponding bacterial vesicular RNAs led to the suppression of IL-8 expression and inhibition of the innate immune response, contributing to the persistence of microorganisms. Earlier, we showed that EVs from A. laidlawii also contain short RNAs, including homologous tRNAMet [9]. However, no significant changes in the expression of IL-8, as well as another critical proinflammatory cytokine, IL-6, were detected in our study upon infection of HSF with the vesicles of mycoplasma (22.71 ± 0.89 and 19.69 ± 2.86 pg/mL in the control and experiment for IL-8, p < 0.05; 11.14 ± 0.22 and 11.42 ± 0.78 pg/ml in the control and experiment for IL-6, p < 0.05).

The time and level of the changes in cytokine expression can vary significantly depending on the medium, cell line, bacterial strain producing vesicles, and the quantitative ratio between eukaryotic cells and bacterial vesicles. The time-dependent response of human fibroblasts to the internalization of A. laidlawii cells and expression of cytokines has still not been studied. There is only one known study [12] devoted to the analysis of time-dependent changes in the transcriptomic profile of human cells (HeLa) during internalization and persistence of mycoplasma cells (Mycoplasma hominis). In this study, significant changes in the expression of cytokine genes were revealed 4 and 48 h after the initiation of the co-incubation of bacterial and eukaryotic cells: in both cases, the IL-6 gene turned out to be in the stress-reactive pool, but not IL-8. In this regard, we analyzed the samples not only 4 h but also 48 h after the initiation of the co-incubation of mycoplasma EVs and HSF. A statistically significant change in the expression of IL-6 was detected (6.42 ± 0.6 and 5.13 ± 0.28 pg/mL in the control and experiment, respectively; p < 0.05), but not in the expression of IL-8 (8.59 ± 3.23 and 17.64 ± 5.88 pg/mL in the control and experiment, respectively; p < 0.05). The data obtained by us attest to the differences in the molecular mechanisms for the induction of immunocompromise in mycoplasmas and classical bacteria.

Tolerance of the innate immunity associated with the lack of any operational modulation of the expression of proinflammatory cytokines (IL-6 and IL-8) does not cancel cellular reactivity to the infectious agent: the molecular signature of the infection can be detected using modern high-resolution methods, including variants of immune electron microscopy, as well as omics profiling [13]. Having used a combination of 2D-DIGE and MALDI-TOF/TOF (the proteomic analysis technology based on the application of the two-dimensional gel electrophoresis of polypeptides stained with different (in the control and experiment, respectively) fluorescent dyes), followed by the identification of differentially presented proteins using MALDI-TOF/TOF and the Mascot software in the mode of Peptide Mass Fingerprint, as well as the DAVID functional annotation tool, we found that a HSF infection with the extracellular vesicles of A. laidlawii leads to a modulation of the fibroblast proteome: changes in the protein representation are recorded 4 h after the initiation of the incubation of mycoplasma EVs with eukaryotic cells; i.e., before any changes in the secretion of IL-6 and IL-8 cytokines are detected. Differentially expressed fibroblast proteins (deposited by us in the ProteomeXchange database, No. PXD027040) are involved in the folding, cytoskeleton formation, biogenesis of EVs (exosomes and microvesicles), immunoreactivity, and cell proliferation. Most of the identified proteins are stress-reactive: they can participate in the cellular response to bacterial and/or viral infections [14]. Among those, there are proteins that are associated with both a positive and negative regulation of apoptosis (TERA, LEG1 and ENPL, CH60, ANXA5, GRP78, HSPB1, CRYAB, respectively).

**Fig. 3:**
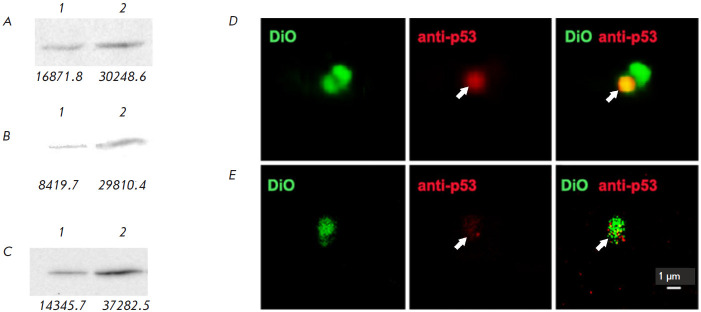
Detection of proteins in human skin fibroblasts using Western blotting (*A–C*) and p53 in HSF-derived microvesicles by confocal laser scanning microscopy (*D*, *E*). Western blotting of cellular proteins with anti-β-actin (*A*), anti-p53 (*B*), and anti-HSP7C (*C*) antibodies. Lanes 1 and 2: proteins of fibroblasts incubated without and with the EVs of *A. laidlawii*, respectively. The intensity of the protein bands is determined by the ImageJ software and is indicated below the lanes. Protein p53 in the microvesicles produced by human skin fibroblasts incubated with (*D*) and without (*E*) EVs of *A. laidlawii*. Arrows indicate the p53 signal

The known limitations of the 2D-DIGE and MALDI-TOF/TOF proteomic analysis variants (low-copy-number proteins cannot be visualized when gels are stained; high-copy-number proteins overlap and hide nearby spots; strongly alkaline proteins are poorly isoelectrofocused; high-molecular-weight proteins do not pass through the pores of the gels used; low-molecular-weight proteins cannot be effectively separated; hydrophobic proteins do not dissolve in the buffer used), including with respect to the detection of a pool of differentially expressed proteins in the eukaryotic cell (the ratio of the visualized, analyzed, reported, and theoretical proteomes differs significantly from the ratio in bacterial cells with a small genome, which is optimal for the relevant studies) [15, 16] determine its limits: not all proteins differentially expressed in a eukaryotic cell can be detected using global proteomic profiling. In this regard, in order to assess the expression of the relevant specific proteins that are not included in the identified stress-reactive pool, one needs to conduct additional targeted analyses (e.g., using Western blotting, which is also recommended for validating global proteomic profiling data [17]). Since p53, the key player in the outcome of pro-and anti-apoptotic processes, was not found within the pool of the identified proteins [18], we conducted a targeted analysis of the representation of this protein in the fibroblasts, as well as in the extracellular vesicles secreted by the fibroblasts (Fig. 3). According to our findings, infection of human skin fibroblasts with A. laidlawii vesicles increases the amount of p53 in cells and does not suppress the secretion of the protein: p53 is found in the extracellular vesicles derived from the fibroblasts of both the control and experimental samples.

The extracellular vesicles of eukaryotes include different groups of vesicles secreted into the extracellular space, which differ in function, size, composition, and biogenesis: exosomes, microvesicles, and apoptotic bodies. Microvesicles form upon protrusion of the plasma membrane; the diameter of these structures is 100–1,000 nm, and their density is 1.25–1.30 g/mL. Apoptotic bodies are released from the plasma membrane of cells at the late stage of apoptosis; the diameter of these structures is 1–5 µm, and their density is 1.18–1.28 g/mL. Exosomes form inside cells from late endosomes known as multivesicular bodies (when a late endosome merges with the plasmalemma, the exosomes find themselves outside the cell); the diameter of these structures is 30–150 nm, and their density is 1.13–1.21 g/mL [19, 20]. The difference in the size and density of vesicles belonging to the various groups is responsible for the likelihood of their differentiation: apoptotic bodies can be distinguished from smaller sized EVs (exosomes and microvesicles) by microscopy, while exosomes and microvesicles can be separated at the stages of ultracentrifugation and Optiprep density gradient ultracentrifugation because of the difference in their size and density. In the centrifugation mode used in our study, exosomes are not precipitated to the test tube bottom and lie higher in the density gradient than microvesicles. High-resolution microscopy visualization of the samples allows one to determine the size of these structures. No structures whose size would correspond to that of apoptotic bodies were found among our preparations: the diameter of individual vesicles isolated from the eukaryotic cells in the studied samples lay in the range of 200–800 nm, which indicates that they could belong to the group of microvesicles.

## CONCLUSIONS

We have shown that EVs from A. laidlawii, a ubiquitous mycoplasma that is the main contaminant of cell cultures, are able to penetrate into eukaryotic cells in vitro and modulate the cellular proteome. The molecular mechanisms behind the interaction of mycoplasma vesicles with eukaryotic cells and the contribution of the corresponding nanostructures to the molecular machinery of cellular permissivity have yet to be clarified. Elucidation of these mechanisms is important both for fundamental research into the simplest prokaryotes and for the practical development of mechanisms to control hypermutable bacteria that infect humans, animals and plants, as well as contaminate cell cultures and vaccine preparations.

## Abbreviations

EVs: extracellular vesicles
HSF: human skin fibroblast
2D-DIGE: two-dimensional difference gel electrophoresis
MALDI-TOF/TOF: matrix-assisted laser desorption/ionization time-of-flight/ time-of-flight
PBS: phosphate buffered saline
